# Interleukin-10 Haplotype May Predict Survival and Relapse in Resected Non-Small Cell Lung Cancer

**DOI:** 10.1371/journal.pone.0039525

**Published:** 2012-07-27

**Authors:** Yaw-Cheng Wang, Wen-Wei Sung, Tzu-Chin Wu, Lee Wang, Wen-Pin Chien, Ya-Wen Cheng, Chih-Yi Chen, Shwn-Huey Shieh, Huei Lee

**Affiliations:** 1 Institute of Medicine, Chung Shan Medical University, Taichung, Taiwan, ROC; 2 School of Medicine, Chung Shan Medical University, Taichung, Taiwan, ROC; 3 Department of Internal Medicine, Chung Shan Medical University Hospital, Taichung, Taiwan, ROC; 4 Department of Public Health, Chung Shan Medical University, Taichung, Taiwan, ROC; 5 Division of Preclinical Science, Center for Drug Evaluation, Taipei, Taiwan, ROC; 6 Department of Surgery, China Medical University Hospital, Taichung, Taiwan, ROC; 7 Department of Health Services Management, China Medical University and Hospital, Taichung, Taiwan, ROC; 8 Department of Medical Research, Chung Shan Medical University Hospital, Taichung, Taiwan, ROC; 9 Institute of Medical Sciences, Tzuchi University, Hualien, Taiwan, ROC; National Taiwan University Hospital, Taiwan

## Abstract

IL-10 is associated with tumor malignancy via immune escape. We hypothesized that IL-10 haplotypes categorized by IL-10 promoter polymorphisms at –1082A>G, –819C>T, and –592C>A might influence IL-10 expression and give rise to non-small cell lung cancer (NSCLC) patients with poor outcomes and relapse. We collected adjacent normal tissues from 385 NSCLC patients to determine IL-10 haplotypes by direct sequencing and polymerase chain reaction restriction fragment length polymorphism (PCR-RFLP). Of the 385 tumors, 241 were available to evaluate IL-10 mRNA expression levels by real-time RT-PCR. The influence of IL-10 haplotypes on overall survival (OS) and relapse free survival (RFS) were determined by Kaplan-Meier and multivariate Cox regression analysis. The results showed that IL-10 mRNA levels were significantly higher in tumors with the non-ATA haplotype than with the ATA haplotype (P = 0.004). Patients with the non-ATA haplotype had shorter OS and RFS periods than did patients with the ATA haplotype. This may be associated with the observation that the number of tumor-infiltrating lymphocytes was decreased in the tumors with higher levels of IL-10. Consistently, T cells from the peripheral blood of the patients with non-ATA haplotype were more susceptible to apoptosis and less cytotoxic to tumor cells, compared to those from the patients with ATA haplotype. The results suggest that IL-10 can promote tumor malignancy via promoting T cell apoptosis and tumor cell survival, and IL-10 haplotype evaluated by PCR-RFLP or direct sequencing may be used to predict survival and relapse in resected NSCLC, helping clinicians to make appropriate decisions on treatment of the patients.

## Introduction

Interleukin-10 (IL-10), an important immunoinhibitory cytokine, is part of a balanced network of cytokines [1]–[5]. The IL-10 cytokine is produced by several cells including normal and neoplastic B cells, stimulated monocytes, macrophages, and a subset of T cells [1]–[4]. Many case-control studies have indicated an association of IL-10 promoter polymorphisms (SNPs) with human cancer risks, including risk of lung cancer [6]–[9]. Of the IL-10 promoter SNPs, ones at −1082A>G, −819C>T, and −592G>A have been the focus of recent studies, and the phenotypes of these single nucleotide SNPs have been further confirmed by functional assays in cell models [10], [11].

Tumor immune surveillance studies have revealed an association between IL-10 and the development of human cancers such as large B-cell lymphoma, T-cell non-Hodgkin lymphoma, and colon, prostate, breast, gastric, myeloma, and lung cancers [4], [6]–[9], [12]–[22]. In lung cancer cases, some reports have indicated that loss of IL-10 in lung tumors may promote tumor progression and result in poor clinical outcomes in patients; however, an opposite effect has been reported in other studies [23]–[29]. Interestingly, the absence of IL-10 expression has been associated with poor outcome in stage I non-small cell lung cancer (NSCLC) [24], [25], while in late-stage NSCLC, the presence of IL-10-positive macrophages at the tumor margins can be an indicator of poor prognostic outcome [23]. In addition, shorter survival times have been reported in advanced lung cancer patients who had high serum IL-10 levels, when compared with similar patients who had low serum IL-10 levels [26]. A clear role for IL-10 in lung tumorigenesis therefore remains to be identified.

**Figure 1:**
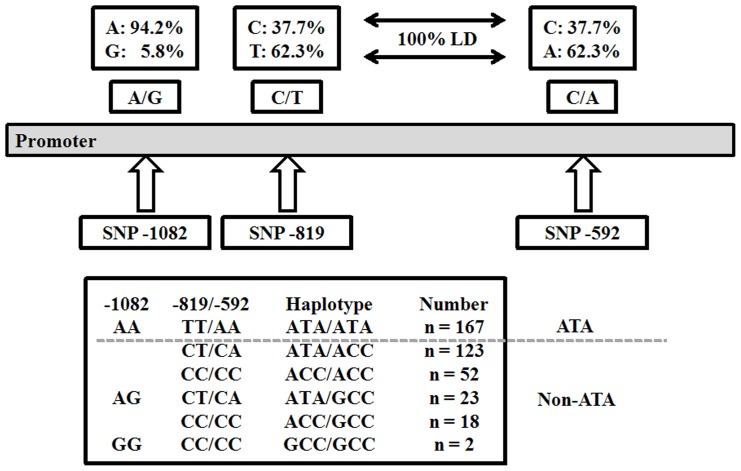
Allele/haplotype frequencies of IL-10 SNPs. SNPs at positions −819 and −592 in the IL-10 promoter were in complete linkage disequilibrium. Three IL-10 haplotypes were observed and the occurrence of each haplotype or haplotype combination is listed in the shaded boxes.

In the present study, we examined normal lung tissues adjacent to surgically resected NSCLC tumors in 385 patients in order to identify IL-10 promoter SNPs at −1082A>G, −819C>T, and −592C>A by direct sequencing and polymerase chain reaction restriction fragment length polymorphism (PCR-RFLP). These three IL-10 promoter SNPs have been reported to produce mainly three haplotypes: GCC, ACC, ATA [30]–[33]. In the present study, patients were categorized into two haplotypes, ATA and non-ATA (Fig. 1), which had been used in two previous reports [34], [35]. We questioned: 1) whether tumors from non-ATA carriers have higher IL-10 mRNA expression levels than tumors from ATA carriers, 2) whether patients with a non-ATA haplotype or higher IL-10 mRNA levels in lung tumors have greater tumor immune surveillance, and 3) whether IL-10 haplotype or mRNA expression could be used to predict overall survival (OS) and relapse free survival (RFS) in resected NSCLC patients.

**Table 1:** Correlations between IL-10 haplotypes and clinical characteristics in 385 non-small cell lung cancer patients.

		IL-10 haplotype		
Parameter	Case no.	ATA	Non ATA	P value^1^
Age				
<65	163	73 (44.8)	90 (55.2)	0.633
≥65	222	94 (42.3)	128 (57.7)	
Gender				
Female	117	58 (49.6)	59 (50.4)	0.105
Male	268	109 (40.7)	159 (59.3)	
Smoking status				
Nonsmoker	197	95 (48.2)	102 (51.8)	0.049
Smoker	188	72 (38.3)	116 (61.7)	
Stage				
Ι and II	212	94 (44.3)	118 (55.7)	0.673
III	173	73 (42.2)	100 (45.9)	
T classification				
1 and 2	290	121 (41.7)	169 (58.3)	0.253
3 and 4	95	46 (48.4)	49 (51.6)	
N classification				
0	185	92 (49.7)	93 (50.3)	0.016
1 and 2	200	75 (37.5)	125 (62.5)	
Tumor type				
SQ	191	85 (44.5)	106 (55.5)	0.658
AD	194	82 (42.3)	112 (57.7)	
Disease relapse				
Negative	229	99 (43.2)	130 (56.8)	0.954
Positive	124	54 (43.5)	70 (56.5)	

SQ, squamous cell carcinomas; AD, adenocarcinomas.

1 Two sided Chi-square test.

**Figure 2:**
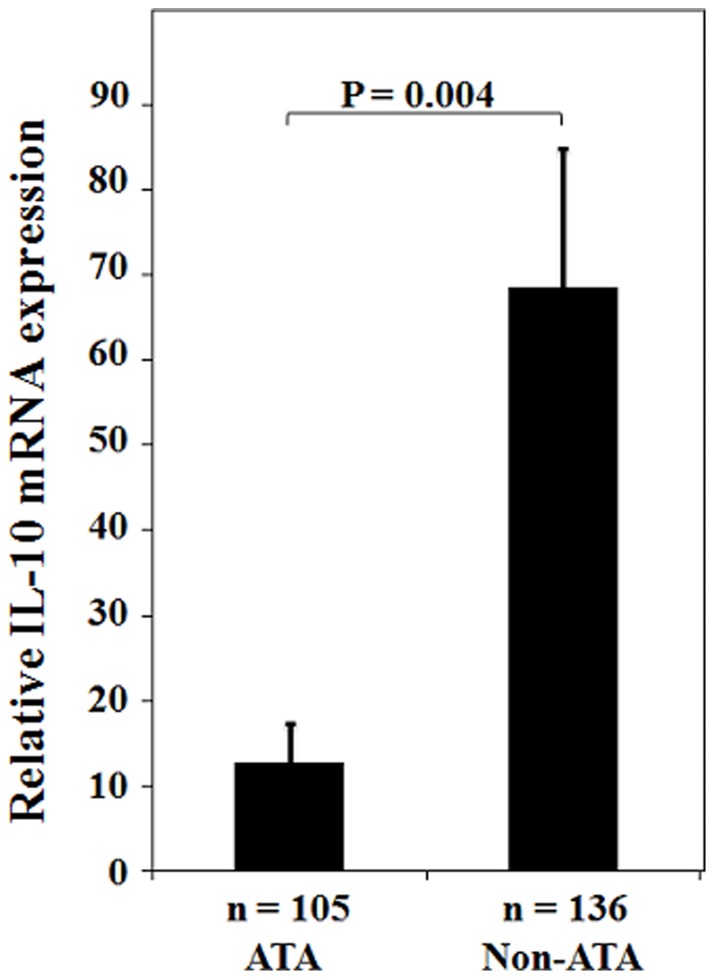
IL-10 mRNA expression (mean ± standard error) in tumor tissue by promoter haplotypes.

**Figure 3:**
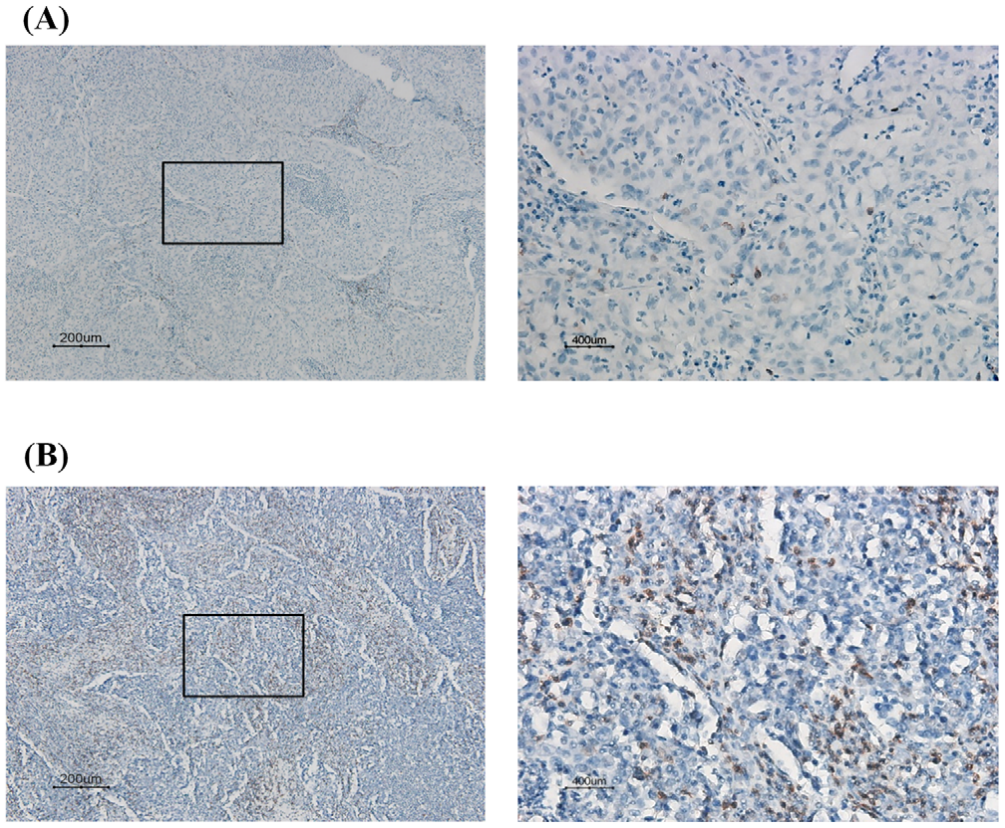
Representative immunostaining of CD3^+^ TILs in lung tumors. (A) Representative of CD3 immunostaining of TILs in lung tumors with low TIL density (<25 CD3^+^/HPF) (left: 100x; right: 400x); (B) TILs presented in lung tumors show high TIL density (≥25 CD3^+^/HPF) (left: 100x; right: 400x).

**Table 2:** Correlations between tumor infiltrating lymphocyte counts and IL-10 haplotype in 87 non-small cell lung cancer patients.

		TIL (count/HPF)		
IL-10 haplotype	Case no.	<25	≥25	P value^1^
All cases				
ATA	36	18 (50.0)	18 (50.0)	0.236
Non-ATA	51	32 (62.7)	19 (37.3)	
Early stage				
ATA	15	7 (46.7)	8 (53.3)	0.988
Non-ATA	28	13 (46.4)	15 (53.0)	
Late stage				
ATA	21	11 (52.4)	10 (47.6)	0.032
Non-ATA	23	19 (82.6)	4 (17.4)	

1 Two sided Chi-square test.

**Figure 4:**
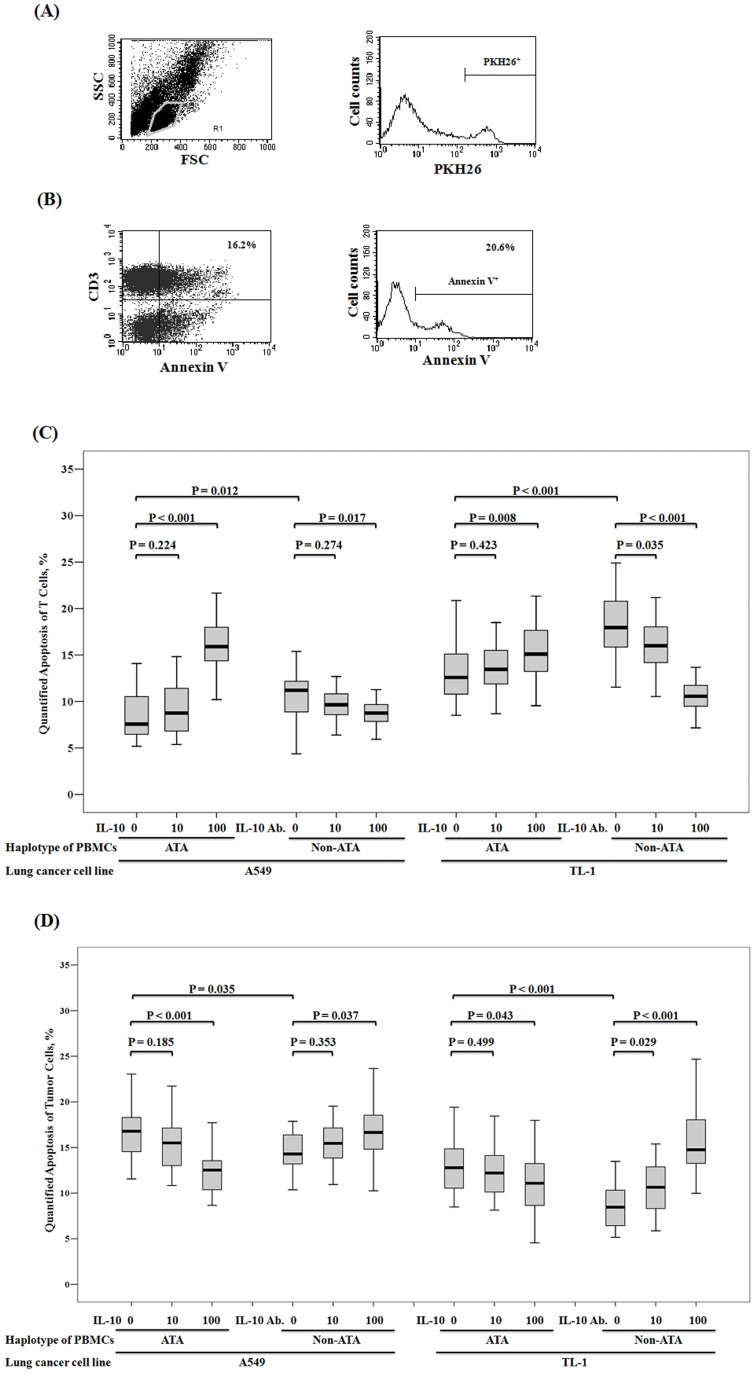
Greater T cell apoptosis resulting in reduced tumor cell apoptosis after co-culture with PBMCs carrying the IL-10 non-ATA haplotype versus the IL-10 ATA haplotype. T cell apoptosis was defined as Annexin V^+^/CD3^+^ and tumor cell apoptosis was defined as Annexin V^+^/PKH26^+^. (A) Representative flow cytometry of SSC-FSC scale for gating lymphocyte population, and PKH26 stating for marking tumor cells. (B) Representative flow cytometry of apoptosis result of CD3^+^ T cells and PKH26 gated tumor cells. (C) Higher level of T cell apoptosis after co-culture with tumor cells from 22 healthy male volunteers that harbored the IL-10 non-ATA haplotype versus the IL-10 ATA haplotype (T cell apoptosis rates during co-culture with A549: 10.49±3.04 vs. 8.34±2.37, P = 0.011; during co-cultured with TL-1: 18.30±3.82 vs. 12.97±2.94, P<0.01). We added IL-10 recombinant protein to the co-culture medium of PBMCs harboring the IL-10 ATA haplotype, and the T apoptosis rate was increased (increased 7.63% during co-culture with A549, P<0.001; increased 2.73% during co-culture with TL-1, P = 0.008). We also added IL-10 neutralized antibody to the co-culture medium of PBMCs harboring the non-ATA IL-10 haplotype, and the T apoptosis rate was decreased (by 1.78% during co-culture with A549, P = 0.017; by 7.75% during co-culture with TL-1, P<0.001). (D) Higher level of tumor cell apoptosis after co-culture with PBMCs from 22 healthy male volunteers that harbored the IL-10 ATA haplotype versus the non-ATA haplotype (apoptosis of A549: 16.80±3.38 vs. 14.45±3.78, P = 0.035; apoptosis of TL-1: 12.95±3.05 vs. 8.68±2.57, P<0.01). We added IL-10 recombinant protein to the co-culture medium of PBMCs harboring the IL-10 ATA haplotype, and the tumor apoptosis rate was decreased (decreased 4.29% of A549, P<0.001; decreased 1.94% of TL-1, P = 0.043). We also added IL-10 neutralized antibody to the co-culture medium of PBMCs harboring the IL-10 non-ATA haplotype, and the tumor apoptosis rate was increased (increased 2.46% of A549, P = 0.037; increased 6.67% of TL-1, P<0.001).

**Figure 5:**
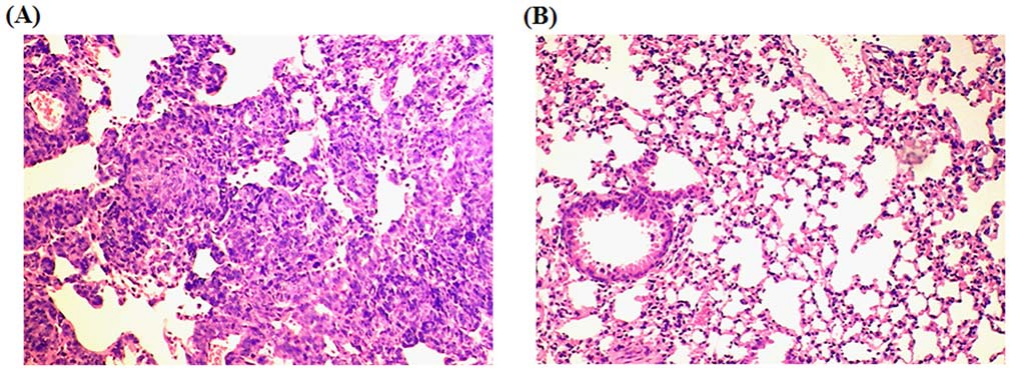
Histological examination of TC-1 tumors in lung following i.v. injection of 1×10^5^ TC-1 cells with injection of IgG antibody or IL-10 neutralize antibody (**20μg per 3 days**)**.** Mice were sacrificed in the 14^th^ days. Lung metastasis was found in mice injected with IgG antibody (A) and was not found in those with IL-10 neutralize antibody (B).

**Figure 6:**
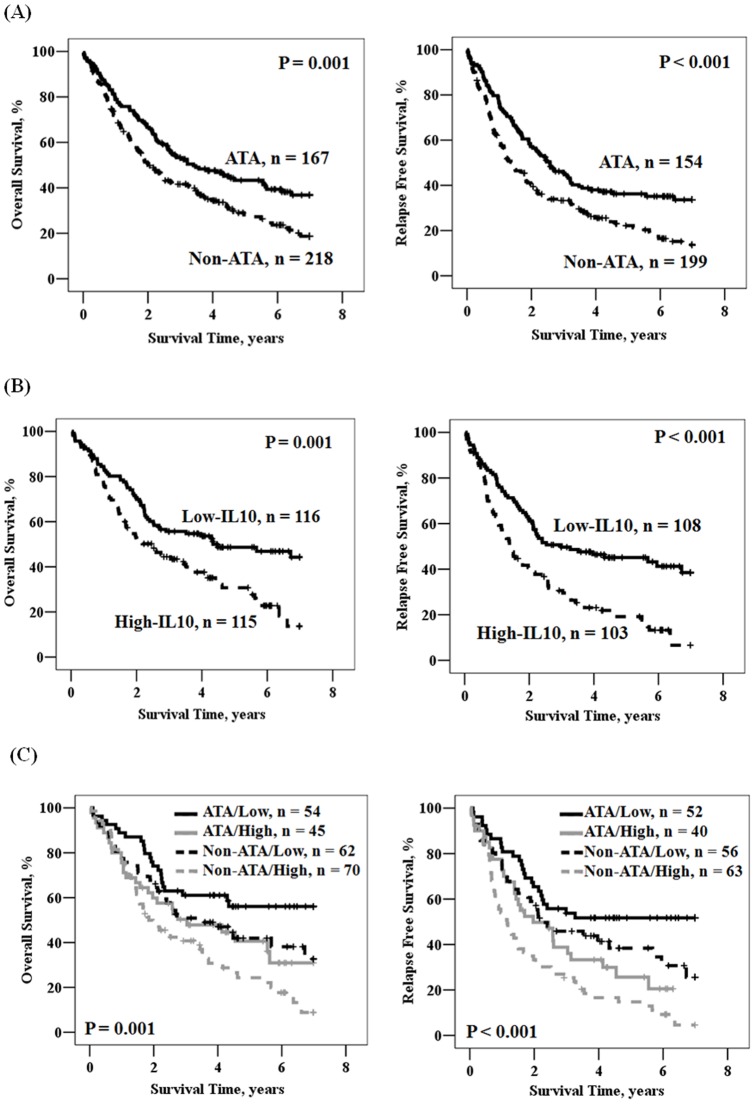
Kaplan-Meier actuarial analysis of RFS and OS. According to IL-10 haplotype (A), IL-10 mRNA (B), and the combination of IL-10 haplotype and mRNA (C).

**Table 3:** Multivariate analysis of the influence of IL-10 haplotype determined from IL10 mRNA on overall survival and relapse free survival in non-small cell lung cancer patients.

Parameter	OS						RFS					
	Case no.	Median Survival, Month	5-year Survival, %	HR	95% CI	P	Case no.	Median Survival, Month	5-year Survival, %	HR	95%CI	P
**IL-10 haplotype**												
ATA	167	40.9	43.3	1.000	Referent		154	30.9	36.2	1.000	Referent	
Non-ATA	218	24.1	28.2	1.431	1.104–1.856	0.007	199	16.8	22.2	1.556	1.200–2.018	<0.001
**IL-10 mRNA**												
Low	116	52.1	48.7	1.000	Referent		108	35.9	45.1	1.000	Referent	
High	115	30.2	30.7	1.553	1.081–2.174	0.017	103	17.2	19.2	1.755	1.245–2.473	0.001
**Haplotype/mRNA**												
ATA/Low	54	83.8	56.1	1.000	Referent		52	83.8	51.7	1.000	Referent	
Non-ATA/High	70	22.2	24.3	1.892	1.163–3.077	0.010	63	13.9	14.8	2.344	1.456–3.776	<0.001

Adjusted for age, gender, smoking, tumor type, and stage.

## Materials and Methods

### Patients

This study included 385 patients with NSCLC. All patients were unrelated ethnic Chinese and residents of central Taiwan. Patients had been diagnosed with adenocarcinoma (194; 50.4%) or squamous cell carcinoma (191; 49.6%) and underwent surgical resection at the Division of Thoracic Surgery, Taichung Veterans General Hospital, between 1993 and 2004. Samples were immediately frozen at surgery and kept at −80°C until processed. The study was approved by the Institutional Review Board (Institutional Review Board, Chung Shan Medical University Hospital. CSMUH No: CS11177). Cancer relapse data were obtained by chart review and confirmed by thoracic surgeons. Clinical parameters and OS and RFS data were collected from chart reviews (32 patients had no relapse data) and the Taiwan Cancer Registry, Department of Health, Executive Yuan, ROC.

### Genomic DNA extraction, RNA extraction, and cDNA synthesis

Genomic DNA was extracted by conventional methods. Surgically resected normal tissues adjacent to the lung tumor were prepared by using proteinase K digestion and DNA was extracted using phenol-chloroform, followed by ethanol precipitation.

Total RNA was extracted from 241 available lung tumor tissues using TRIzol reagent (Invitrogen). First-strand cDNA synthesis in the presence of random primers was performed using a high-capacity cDNA reverse transcription kit (Applied Biosystems) according to the manufacturer's instructions.

### PCR-RFLP analysis for IL-10 -592C/A genetic SNP

Genotypes of IL-10 -592C/A were determined by PCR-RFLP as described by Rad et al. [36]. PCR amplification products from 50 samples were randomly selected for direct sequencing to confirm the genotype indicated by PCR-RFLP.

### Direct sequencing for IL-10 -1082A/G and -819C/T genetic SNPs

SNPs of IL-10 -1082G/A and -819C/T were determined by direct sequencing of PCR products amplified from the DNA of normal tissues adjacent to the tumors. The DNA samples were prepared using proteinase K digestion and phenol-chloroform extraction, followed by ethanol precipitation. Primers used for DNA amplification and direct sequencing were: 5′-CTCGCCGCAACCCAACTGGC-3′ (F) and 5′-TGGGGGAAGTGGGTAAGAGT-3′ (R). The PCR cycle conditions consisted of an initial denaturation step at 94°C for 10 min, followed by 35 cycles of 30s at 94°C; 45s at 56°C; 45s at 72°C; and a final elongation at 72°C for 10 min. The PCR products were sequenced using an Applied Biosystems 3100 Avant Genetic Analyzer (Applied Biosystems).

### Real-time RT-PCR

Real-time RT-PCR amplification of cDNA samples was performed with an ABI 7500 Real time PCR System (Applied Biosystems) and SYBR Green dye to quantify IL-10 mRNA transcripts. Real-time RT-PCR primers were as follows: for IL-10 transcripts, 5′-GGCGCTGTCATCGATTTCTT-3′ (forward) and 5′-TGGAGCTTATTAAAGGCATTCTTCAC-3′ (reverse); for 18S gene transcripts, 5′-TCGGAACTGAGGCCATGA-3′ (forward) and 5′-CCGGTCGGCATCGTTTA-3′ (reverse). The products amplified by IL-10 primers were checked by direct sequencing. The amounts of IL-10 mRNA transcripts were quantified relative to the 18S internal control according to the manufacturer's instructions (Applied Biosystems).

### Peripheral blood monocyte isolation and co-culture experiments

Peripheral blood monocytes (PBMCs) from healthy donors were isolated by Ficoll-Paque (GE Healthcare) density-gradient centrifugation as described previously [37]. The PBMCs were used for the determination of cancer cell-induced T cell apoptosis by co-culture with lung cancer cells at a ratio of 40∶1 for 36 hrs with IL-10 neutralize antibody (MAB2171, R&D Systems) or IL-10 recombinant protein (CYT-500, Prospec). The PBMCs were then collected for analysis of T cell apoptosis by flow cytometry.

### Flow cytometry analysis

A flow cytometer (FACSCalibur; BD Biosciences) was used to determine the cell population size and apoptosis percentage. PBMCs were stained with PE-Cy^TM^7 mouse anti-human CD3 (BD Pharmingen) and the CD3 positive cells in the lymphocyte gate were identified as T cells. Annexin V-FITC (BD Pharmingen) was used to mark cell apoptosis. After staining the cells according to the recommended protocol, the samples were analyzed within 1 hour. For T cell apoptosis, we gated the lymphocyte gate in FSC-SSC, and the population with CD3^+^ and Annexin V^+^ was calculated [37].

### Immunohistochemical staining

Immunohistochemical staining to evaluate CD3 expression in lymphocytes was carried out on whole-mount paraffin sections of lung cancer specimens. Anti-CD3 (1/100) polyclonal primary antibody (Santa Cruz Biotechnology) was used. An immunohistochemistry detection kit for *in vitro* diagnostic use (Invitrogen) was used according to the standard protocol. TIL was counted by random systematic selection of fields. At least five different HPFs were counted in each sample which depends on the size of tumor area.

### Animal model

C57Bl/6 mice were maintained in standard mouse facility (specific pathogen free) at Chung Shan Medical University. TC-1 cell line was kindly provided by Dr. TC Wu (John Hopkins, Baltimore, MD) [38]. TC-1 cells were suspended in PBS at a concentration of 10^5^ cells per 100 μl. Each mouse was injected with 10^5^ TC-1 cells by tail vain injection. Mice received intraperitoneal doses of 200 μg of anti-IL-10 antibody (mAb417 R&D Systems) at the first day of injection; alternated with intraperitoneal doses of 20 μg of anti-IL-10 antibody (mAb417 R&D Systems) spaced by3 days. The mice were sacrificed at the 14^th^ days after tumor injection. HE stain was performed to verify the tumor formation among the organs of mice.

### Statistical analysis

The Student's t test and Chi-square test were applied for continuous or discrete data analysis. The associations between IL-10 promoter polymorphisms and patient survival were estimated using the Kaplan-Meier method and assessed using the log-rank test. Potential confounders were adjusted by Cox regression models, with IL-10 promoter polymorphisms fitted as indicator variables. All statistical analyses were done using the SPSS statistical software program (version 13.0) (SPSS, Inc., Chicago, IL). All statistical testing was done using two-sided tests and P values <0.05 were considered to be statistically significant.

## Results

### Non-ATA haplotype is more common in patients with nodal metastasis

IL-10 haplotypes−ATA/ATA (ATA haplotype) and non-ATA/ATA (non-ATA haplotype)−were categorized by IL-10 promoter SNPs at −1082A/G, −819C/T and −592C/A (Fig. 1). Relationships between IL-10 haplotypes and clinicopathological parameters in 365 NSCLC patients were statistically analyzed by a Chi-square test. As shown in Table 1, the non-ATA haplotype occurred more frequently in patients with nodal metastasis (N1 and N2) than with non-nodal metastasis (N0) (62.5% vs. 50.3%, P = 0.016). In addition, a relatively higher prevalence of nonsmokers was seen among patients with the non-ATA haplotype than with the ATA haplotype (61.7% vs. 51.8%, P = 0.049). Therefore, patients with non-ATA haplotype may have more aggressive tumors with a greater tendency towards relapse.

### Higher IL-10 mRNA levels in lung tumors from non-ATA haplotype patients compared with ATA haplotype patients resulted in poorer tumor immune surveillance partially via decreased numbers of tumor infiltrating lymphocytes

Of the tumors of the 385 lung cancer patients, 241 were available for evaluation of IL-10 mRNA expression levels. As shown in Fig. 2, significantly higher IL-10 mRNA levels were found in tumors from non-ATA haplotype patients than from ATA haplotype patients (mean ± SE, 68.51±16.31 vs. 12.85±4.54, P = 0.004). The distribution of tumor infiltrating lymphocytes (TILs) in lung tumors was evaluated by IHC in 87 available lung tumor paraffin sections. In the entire study population, no difference was found in number of TILs in lung tumors of ATA and non-ATA patients (Table 2, Fig. 3; 50.0 vs. 37.5, P = 0.236). However, a difference in TIL numbers between ATA and non-ATA carriers was observed in late-stage patients (P = 0.032, Table 2), but not in early-stage patients (P = 0.988, Table 2). These results suggest that non-ATA patients who have lower TIL numbers compared with ATA patients may have poorer tumor immune surveillance.

### IL-10 reduces tumor cell apoptosis via increased T cell apoptosis

To verify the possibility of changes in tumor immune surveillance, PBMCs were collected from 22 healthy donors with the ATA haplotype and 22 healthy donors with the non-ATA haplotype. IL-10 mRNA levels in PBMCs from non-ATA haplotype healthy donors were higher than from ATA haplotype healthy donors (mean ± SE, 78.54±13.18 vs. 46.37±3.91, P = 0.028). These cells were then co-cultured with A549 and TL-1 lung cancer cells. The cytotoxicity of lung cancer cells was determined by a flow cytometry with Annexin V^+^/PKH26^+^ and T cell apoptosis was determined with Annexin^+^/CD3^+^. The T cell apoptosis after co-culture with A549 and TL-1 cells was lower in PBMCs from the ATA haplotype healthy donors than in PBMCs from the non-ATA haplotype healthy donors (8.34±2.37 vs. 10.49±3.04, P = 0.012 for A549; 12.97±2.94 vs. 18.30±3.82, P<0.001; Fig. 4A). Consequently, the number of apoptotic tumor cells (A549 and TL-1) was significantly higher after co-culture with PBMCs from the ATA haplotype healthy donors than from the non-ATA healthy donors (16.80±3.38 vs. 14.45±3.78, P = 0.035 for A549; 12.95±3.05 vs. 8.68±2.57, P<0.001; Fig. 4B). To verify whether IL-10 was responsible for this apoptosis of T cells and lung cancer cells, the numbers of apoptotic T cells of PBMCs from the ATA haplotype healthy donors was increased by treatment with 100 ng/ml IL-10 (8.34±2.37 vs. 15.97±3.03, P<0.001 for A549; 12.97±2.94 vs. 15.70±3.54, P = 0.008 for TL-1; Fig. 4A). Conversely, T cell apoptosis in PBMCs from non-ATA healthy donors was markedly decreased in a dose-dependent manner by IL-10 neutralized antibody (Fig. 4A). Consequently, the apoptosis of both lung cancer cell types was decreased by treatment with IL-10 and increased by IL-10 neutralized antibody (Fig. 4B). Collectively, PBMCs from non-ATA carriers had lower apoptotic effects on lung cancer cells than did PBMCs from ATA carriers, and IL-10 was responsible for tumor cell apoptosis after co-culture with PBMCs. These results clearly indicated that IL-10 reduced tumor cell apoptosis via increased T cell apoptosis.

In our animal experiment, TC-1 cells that did not express IL-10 (kindly provided by Dr. T. C. Wu, John Hopkins, Baltimore, MD, USA) were injected into C57BL6 mice via the tail vein. After 14 days, tumors were formed in the whole lung (Fig. 5). However, no tumors were seen after the TC-1 cells treated mice were treated with IL-10 neutralized antibody. These results seem to support previous studies indicating that IL-10 secreted from immune cells may promote lung tumor progression [39].

### Patients with non-ATA haplotype or high IL-10 mRNA levels had poorer outcomes compared with patients with ATA haplotype or low IL-10 mRNA levels

Kaplan-Meier and multivariate Cox regression analyses were conducted to verify whether poorer survival and greater relapse would be seen in patients with the non-ATA haplotype or high IL-10 mRNA levels in lung tumors compared with patients with the ATA haplotype or low IL-10 mRNA levels. The survival curves predicted by Kaplan-Meier analysis showed that patients with the non-ATA haplotype had shorter OS and RFS periods than patients with the ATA haplotype (P = 0.001 for OS, left panel of Fig. 6A; P<0.001 for RFS, right panel of Fig. 6A). Consistent with the prognostic value of the IL-10 haplotype, elevated IL-10 mRNA levels were also observed in a subset of this study population (n = 241), showing that tumors with high IL-10 mRNA levels were correlated with worse OS and RFS than in patients whose tumors had low IL-10 mRNA levels (P = 0.001 for OS, left panel of Fig. 6B; P<0.001 for RFS, right panel of Fig. 6B). As expected, the worst OS and RFS were seen in patients with a non-ATA haplotype and high IL-10 mRNA levels (Fig. 6C). These results suggest that the IL-10 haplotype or IL-10 mRNA expression in lung tumors could be used to predict patients' outcomes.

Multivariate Cox regression was used to explore whether IL-10 haplotype or mRNA levels may independently predict patient's outcome, after adjusting for various parameters including age, gender, smoking status, tumor histology, and cancer stage. As shown in Table 3, patients with the non-ATA haplotype had HRs of 1.431 and 1.556 for shorter OS and RFS, respectively, compared with patients with ATA haplotype (95% CI, 1.104–1.856, P = 0.007 for OS; 1.200–2.018, P<0.001 for RFS). The median durations for OS and RFS in patients with the non-ATA haplotype were 24.1 and 16.8 months, respectively, compared with 40.9 and 30.9 months, respectively, for the ATA haplotype. The 5-year survival rates for patients with non-ATA and ATA haplotypes were 28.2% vs. 43.3% for OS and 22.2% vs. 36.2% for RFS. The independent prognostic value of IL-10 mRNA levels was also shown in a subset of this study population. Patients with high IL-10 mRNA levels had shorter median survival and 5-year survival rate for OS and RFS than patients with low IL-10 mRNA levels (Table 3; 30.2 months vs. 52.1 months, 30.7% vs. 48.7%, P = 0.017 for OS; 17.2 months vs. 35.9 months, 19.2% vs. 45.1%, P = 0.001 for RFS). The HRs of IL-10 mRNA for OS and RFS increased in relation to HRs of IL-10 haplotype (1.553 vs. 1.431 for OS; 1.755 vs. 1.556 for RFS). More interestingly, HRs of patients with non-ATA haplotype plus high IL-10 mRNA level was increased from 1.431 to 1.892 for OS and from 1.556 to 2.344 for RFS when compared with patients with ATA haplotype plus low IL-10 mRNA level. The increase in HRs was not seen in patients with the ATA haplotype plus high IL-10 mRNA or in patients with non-ATA haplotype and low IL-10 mRNA level. These results seem to suggest that IL-10 haplotype or IL-10 mRNA level may independently predict survival and relapse in resected NSCLC patients.

## Discussion

The SNPs of IL-10 at −1082 G>A, −819 C>T, and −592 C>A are GCC, ACC, and ATA haplotypes, respectively [36], [40]. The expression of IL-10 mRNA in peripheral blood cells is highest in individuals with one or two GCC haplotypes, followed by ACC/ACC carriers, and then ATA/ACC and ATA/ATA carriers [36], [40], [41]. In the present study, non-ATA and ATA haplotypes were categorized by the three GCC, ATA, and ACC haplotypes of IL-10 promoter (Fig. 1). A poor prognostic value for OS and RFS was found for the GCC, ACC, and non-ATA carriers (Table S1). However, the prognostic significance of the three carriers was only revealed in late-stage patients, not in early-stage patients of this study population (Table S1). On the other hand, IL-10 mRNA expression levels were significantly higher in carriers with ≥1 copy number of GCC than in carriers without GCC (IL-10 mRNA for GCC vs. non-GCC: 147.90±56.80 vs. 31.72±7.91, P<0.001). No difference in IL-10 mRNA expression was seen for ACC carriers (IL-10 mRNA for ACC vs. non-ACC: 57.69±15.43 vs. 31.15±11.36, P = 0.166). This was consistent with a previous report indicating that the GCC haplotype carriers had a higher lung cancer risk than other carriers [6]. In addition, IL-10 mRNA level was higher in non-ATA haplotype than in ATA haplotype (Fig. 2). Therefore, in the present study, IL-10 haplotypes were assessed for their prognostic significance in NSCLC patients.

The association between IL-10 and tumor progression has frequently been explained by changes in immune suppression and tumor immune surveillance [1]–[4], [42]. In the present study, consistent findings were seen in *in vitro* experiments with PBMCs co-cultured with lung cancer cells and in *in vivo* observations of TILs in tumor tissues (Fig. 4). Previous studies using transgenic mice expressing IL-10 showed that these mice developed larger tumors than did control mice. This result suggests that IL-10 prevents effective immune responses against tumor progression [43].

Previous reports have shown that IL-10 has different prognostic significance in early- and late-stage lung cancer patients [23]–[25]. In the present study population, poorer OS and RFS were observed in late-stage (III) patients with the non-ATA haplotype than in patients with the ATA haplotype (HR, 1.785, P<0.001 for OS; HR, 2.071, P<0.001 for RFS; Table S1); however, no prognostic value was observed for early-stage (I+II) patients (Table S1). When compared with the entire study population, late-stage patients had higher HRs for OS and RFS (1.785 vs. 1.431 for OS; 2.071 vs. 1.556 for RFS; Table S1). This could be explained by significantly higher IL-10 mRNA levels in late-stage patients with the non-ATA haplotype than with the ATA haplotype (87.26±28.83 vs. 10.87±4.53, P = 0.011); however, the difference between non-ATA and ATA haplotypes was marginally observed in early-stage patients (54.13±18.49 vs. 14.52±7.47, P = 0.050). In addition, the prognostic significance of TILs on OS and RFS was also shown in late-stage patients, not in early-stage patients (P = 0.012 for OS, P = 0.003 for RFS; Table S2). Therefore, we suggest that the non-ATA haplotype, with a higher IL-10 mRNA expression level, may be more important in promoting tumor aggressiveness in late-stage patients than in early-stage patients.

In summary, IL-10 haplotypes can be readily determined from patient blood samples and may be useful in predicting survival and relapse in resectable NSCLC patients. To the best of our knowledge, this is the first report showing that IL-10 haplotypes can be used to predict OS and RFS in NSCLC patients. Therefore, we propose that the haplotypes of IL-10 promoter SNPs may be potential biomarkers for prediction of survival and relapse in resectable NSCLC patients.

## Supporting Information

Table S1 **Multivariate analysis of the influence of IL-10 haplotypes on overall survival and relapse free survival in non-small cell lung cancer patients according to stage.**
(DOC)

Table S2 **Multivariate analysis of the influence of tumor infiltrating lymphocyte counts on overall survival and relapse free survival in non-small cell lung cancer patients.**
(DOC)
