# Supplementary material for: Interleukin-10 Haplotype May Predict Survival and Relapse in Resected Non-Small Cell Lung Cancer
Source: PLoS One. 2012 Jul 27;7(7):e39525. doi: 10.1371/journal.pone.0039525 (PMC3407146; doi:10.1371/journal.pone.0039525)
Supplement: Table S1 — Multivariate analysis of the influence of IL-10 haplotypes on overall survival and relapse free survival in non-small cell lung cancer patients according to stage. (DOC) [file pone.0039525.s001.doc]

Table S1. Multivariate analysis of the influence of IL-10 haplotypes on overall survival and relapse free survival in non-small cell lung cancer patients according to stage.

| Parameter | OS | | | | | |  | RFS | | | | | |
| --- | --- | --- | --- | --- | --- | --- | --- | --- | --- | --- | --- | --- | --- |
|  | Case no. | Median Survival, Month | 5-year Survival, % | HR | 95% CI | P |  | Case no. | Median Survival, Month | 5-year Survival, % | HR | 95%CI | P |
| **All case** |  |  |  |  |  |  |  |  |  |  |  |  |  |
| **Haplotype ATA** |  |  |  |  |  |  |  |  |  |  |  |  |  |
| ATA | 167 | 40.9 | 43.3 | 1.000 | Referent |  |  | 154 | 30.9 | 36.2 | 1.000 | Referent |  |
| Non-ATA | 218 | 24.1 | 28.2 | 1.431 | 1.104-1.856 | 0.007 |  | 199 | 16.8 | 22.2 | 1.556 | 1.200-2.018 | <0.001 |
| **Haplotype ACC** |  |  |  |  |  |  |  |  |  |  |  |  |  |
| Non-ACC | 192 | 34.8 | 40.2 | 1.000 | Referent |  |  | 177 | 27.0 | 33.8 | 1.000 | Referent |  |
| ACC | 193 | 26.8 | 29.0 | 1.343 | 1.042-1.730 | 0.023 |  | 176 | 18.0 | 22.5 | 1.390 | 1.078-1.791 | 0.011 |
| **Haplotype GCC** |  |  |  |  |  |  |  |  |  |  |  |  |  |
| Non-GCC | 342 | 33.0 | 36.7 | 1.000 | Referent |  |  | 313 | 25.4 | 29.9 | 1.000 | Referent |  |
| GCC | 43 | 17.2 | 21.7 | 1.774 | 1.084-2.251 | 0.017 |  | 40 | 9.8 | 17.5 | 1.824 | 1.267-2.627 | 0.001 |
| **Early stage** |  |  |  |  |  |  |  |  |  |  |  |  |  |
| **Haplotype ATA** |  |  |  |  |  |  |  |  |  |  |  |  |  |
| ATA | 67 | 66.6 | 51.0 | 1.000 | Referent |  |  | 66 | 38.5 | 42.4 | 1.000 | Referent |  |
| Non-ATA | 82 | 53.5 | 47.1 | 1.018 | 0.624-1.663 | 0.942 |  | 76 | 39.5 | 36.5 | 1.002 | 0.633-1.586 | 0.994 |
|  |  |  |  |  |  |  |  |  |  |  |  |  |  |
|  |  |  |  |  |  |  |  |  |  |  |  |  |  |
| Supplementary table 1. (continued) | | | | | |  |  |  |  |  |  |  |  |
| **Haplotype ACC** |  |  |  |  |  |  |  |  |  |  |  |  |  |
| Non-ACC | 70 | 66.6 | 51.8 | 1.000 | Referent |  |  | 69 | 38.6 | 43.6 | 1.000 | Referent |  |
| ACC | 79 | 53.5 | 46.1 | 0.995 | 0.610-1.622 | 0.982 |  | 73 | 39.5 | 35.0 | 1.002 | 0.633-1.586 | 0.993 |
| **Haplotype GCC** |  |  |  |  |  |  |  |  |  |  |  |  |  |
| Non-GCC | 138 | 80.6 | 48.7 | 1.000 | Referent |  |  | 131 | 38.6 | 39.0 | 1.000 | Referent |  |
| GCC | 11 | 56.0 | 54.5 | 1.326 | 0.573-3.070 | 0.510 |  | 11 | 14.6 | 45.5 | 1.257 | 0.576-2.741 | 0.566 |
| **Late stage** |  |  |  |  |  |  |  |  |  |  |  |  |  |
| **Haplotype ATA** |  |  |  |  |  |  |  |  |  |  |  |  |  |
| ATA | 100 | 31.0 | 38.2 | 1.000 | Referent |  |  | 88 | 24.5 | 31.6 | 1.000 | Referent |  |
| Non-ATA | 136 | 17.6 | 16.5 | 1.785 | 1.305-2.441 | <0.001 |  | 123 | 11.5 | 13.0 | 2.071 | 1.499-2.861 | <0.001 |
| **Haplotype ACC** |  |  |  |  |  |  |  |  |  |  |  |  |  |
| Non-ACC | 122 | 25.6 | 33.6 | 1.000 | Referent |  |  | 108 | 20.0 | 27.5 | 1.000 | Referent |  |
| ACC | 114 | 18.0 | 16.7 | 1.531 | 1.133-2.069 | 0.006 |  | 103 | 12.6 | 13.3 | 1.637 | 1.202-2.229 | 0.002 |
| **Haplotype GCC** |  |  |  |  |  |  |  |  |  |  |  |  |  |
| Non-GCC | 204 | 22.8 | 28.4 | 1.000 | Referent |  |  | 182 | 17.2 | 23.2 | 1.000 | Referent |  |
| GCC | 32 | 15.5 | 11.3 | 1.666 | 1.102-2.517 | 0.015 |  | 29 | 9.8 | 6.9 | 2.139 | 1.399-3.271 | <0.001 |
| *Adjusted for age, gender, smoking, tumor type, and stage. | | | | | | | | | | | | | |
