# Supplementary material for: Interleukin-10 Haplotype May Predict Survival and Relapse in Resected Non-Small Cell Lung Cancer
Source: PLoS One. 2012 Jul 27;7(7):e39525. doi: 10.1371/journal.pone.0039525 (PMC3407146; doi:10.1371/journal.pone.0039525)
Supplement: Table S2 — Multivariate analysis of the influence of tumor infiltrating lymphocyte counts on overall survival and relapse free survival in non-small cell lung cancer patients. (DOC) [file pone.0039525.s002.doc]

Table S2. Multivariate analysis of the influence of tumor infiltrating lymphocyte counts on overall survival and relapse free survival in non-small cell lung cancer patients.

| TIL | OS | | | | | |  | RFS | | | | | |
| --- | --- | --- | --- | --- | --- | --- | --- | --- | --- | --- | --- | --- | --- |
|  | Case no. | Median Survival, Month | 5-year Survival, % | HR | 95% CI | P |  | Case no. | Median Survival, Month | 5-year Survival, % | HR | 95%CI | P |
| All cases |  |  |  |  |  |  |  |  |  |  |  |  |  |
| < 25 | 50 | 17.8 | 20.6 | 1.000 | Referent | 0.083 |  | 48 | 8.5 | 18.5 | 1.000 | Referent | 0.037 |
| ≥ 25 | 37 | 42.2 | 24.3 | 0.620 | 0.361-1.064 |  |  | 32 | 31.2 | 32.0 | 0.546 | 0.310-0.964 |  |
| Early stage |  |  |  |  |  |  |  |  |  |  |  |  |  |
| < 25 | 20 | 38.6 | 44.1 | 1.000 | Referent | 0.717 |  | 19 | 38.4 | 42.5 | 1.000 | Referent | 0.751 |
| ≥ 25 | 23 | 42.6 | 40.1 | 0.849 | 0.351-2.055 |  |  | 20 | 38.6 | 30.6 | 0.867 | 0.358-2.096 |  |
| Late stage |  |  |  |  |  |  |  |  |  |  |  |  |  |
| < 25 | 30 | 10.8 | 6.7 | 1.000 | Referent | 0.012 |  | 29 | 7.7 | 3.4 | 1.000 | Referent | 0.003 |
| ≥ 25 | 14 | 28.1 | 17.9 | 0.372 | 0.172-0.805 |  |  | 12 | 19.2 | 33.3 | 0.266 | 0.111-0.636 |  |
| *Adjusted for age, gender, smoking, tumor type, and stage. | | | | | | | | | | | | | |
